# Evaluation of the use of health care services for non-communicable disease and prevention by children and adolescents in south Italy

**DOI:** 10.1186/s12913-017-2489-4

**Published:** 2017-08-04

**Authors:** Flora Ascione, Diana Cascone, Francesco Napolitano, Gabriella Di Giuseppe

**Affiliations:** Department of Experimental Medicine, University of Campania Luigi Vanvitelli, Via Luciano Armanni, 5, 80138 Naples, Italy

**Keywords:** Children, Cross-sectional study, Health care services use, Italy, Multivariate regression analysis

## Abstract

**Background:**

The objectives of this investigation are to evaluate the use of health care services for non-communicable disease and prevention by children and adolescents and to identify the factors linked to the use of health care services.

**Methods:**

This cross-sectional survey was conducted between December 2014 and January 2015 among 1198 parents of students aged between 5 and 18 years attending 12 selected schools in the geographic area of Salerno and Naples, Italy, using a self-administered questionnarie.

**Results:**

68.2% of parents stated that had visited their general practitioner (GP) or family pediatrician (FP) with their child in the last year. 66.2% of children had had at least one visit to a specialist and more than half (54.8%) had had preventive care visits in the last year. The use of preventive care visits within last year was significantly higher amongst female, among those who had visited their GP or FP and among those who had a parent with a college degree or higher. The proportion of emergency department visits and hospital admissions reported were 12.8% and 4.7% respectively.

**Conclusion:**

This results highlights the need of educational interventions for parents and adolescents in order to increase the utilization of preventive health services.

**Electronic supplementary material:**

The online version of this article (doi:10.1186/s12913-017-2489-4) contains supplementary material, which is available to authorized users.

## Background

Noncommunicable diseases (NCDs) cause millions of deaths each year and are responsible for over two thirds of all deaths worldwide. For this reason the Global action plan for the prevention and control of NCDs 2013–2020 has set the target of reducing by 25% the relative risk of premature mortality from these diseases by 2025 [1].

It is well known that NCDs in adulthood begin in the younger ages, and the main causes of illness and death in the population can be considered largely preventable through accessible preventative actions and changes in the lifestyles of children and adolescents [2–5]. Childhood is a time of rapid growth and change and since children have more visits in their early years, even if a child is healthy, the visits in this period are a good time to focus on the child’s wellbeing and on prevention of diseases [6]. Indeed, the prevention of health problems helps keep a healthy child enabling them to become a healthy adult.

To improve and preserve the quality of health for children and adolescents, it is essential to have an ordinary and appropriate utilization of the health care services, which provide correct treatment and health promotion. These services include parental health education, routine ceck-ups, immunizations and preventive care visits. The use of the health care services may be affected by many factors including structural and organizational characteristics of services [7–11]; in Italy, in particular, a country with a system of universal health coverage, the use of the health care services are guaranteed and provided free of charge throught the family pediatricians (FP) or general practitioners (GP). GPs and FPs represents the gatekeepers in the utilization of the all services and benefits of the Italian National Health Service [12].

In published literature, several studies have investigated the use and the associated factors to health care services in children and young adults [7, 13–22] while there are few investigations regarding the utilization of preventive health services [23–26] and no research has been conducted in Italy on this issue.Therefore, it seemed interesting to evaluate the use of the health care services in a sample of children and adolescents for the treatment and prevention of their diseases and to identify the factors linked to these outcomes of interest.

## Methods

This cross-sectional survey was conducted between December 2014 and January 2015 among 1198 parents of students aged between 5 and 18 years attending 12 selected schools in the geographical areas of Salerno and Naples, Italy. The survey design included a two-stage cluster sampling procedure. Specifically, in the frist stage, from the list of public schools of the geographical areas of Salerno and Naples, three primary and nine secondary schools were randomly selected. In the second stage, from each selected school the students were selected through a simple random sampling.

The sample size was determined based on the assumptions that 78% of children and adolescents had had a health care services use as in accordance with published literature [15, 18], a confidence interval of 95%, a margin of error of 5%, an expected response rate of 50% and a design effect of 2 for a total number of 1196 participants. Considering the total number of the sample size, 12 schools were selected because it was thought to sample circa 100 participants from each school.

Before starting the survey to the directors of each school were sent a letter requesting their cooperation in the survey and in which the objectives of the study and the methods to collect the information were specified. After having obtained approval, the students were given a sealed envelope to take home that contained a cover letter, an informed consent form, a questionnaire and a pre-addressed envelope to return the completed questionnaire to the organizers of the survey. The letter included a full description of the study and its importance and explained that the information would be collected respecting the parents’ privacy and anonymity. It invited only one of the parents to provide the consent to participate and to complete the questionnaire. No payments or gifts were given to the respondents.

The study protocol and the questionnaire were approved by the Ethical Committee of the University of Campania Luigi Vanvitelli. Written informed consent was obtained from each parent.

The questionnaire was composed of three sections: (1) socio-demographical characteristics of the parents (gender, age, marital status, education level, employment status, number and age of children, number of cohabiting family members, partner’s employment status, socio-economic status, having family member’s occupation related to health care). The socio-economic status was evalueted based on the household employment status; (2) socio-demographical and clinical characteristics of the children (gender, age, school, chronic conditions and health status in the last year, the parent’s perception of the children health status); (3) use of health care services in the previous 12 months (reasons and number of GP/FP visits, type, reasons and number of specialist visits, type and number of preventive care visits, reasons and number of visits to the emergency department (ED), reasons and number of hospital admissions). The preventive care visits have been defined as a consultation or periodic physical examination to identify potential health problems in healthy people. The self-administered questionnaire included closed and open-ended types of questions and 5-point Likert scale responses (see Additional file 1).

Before starting the survey, a pilot study was carried out on a sample of 25 parents, which was not included in the final sample, to test the understandability and clarity of the questionnarie questions.

### Statistical analysis

Following a descriptive analysis of the data, the inferential analysis was conducted according to a double stage strategy. In the first stage, four multivariate logistic regression full models were built to evaluate the effect of each independent variable on the following outcomes of interest: utilization of the GP or pediatrician in the last year (Model 1), at least one specialist visit in the last year (Model 2), at least one preventive care visit in the last year (Model 3) and at least one emergency department visit in the last year (Model 4). In this stage, the selection of variables included in saturated models was done considering previous investigation in published literature [14, 15, 18, 20], while other variables were chosen because they were considered as interesting predictors of the outcomes. In the second stage, the variables included in the final models were determined using a backwards selection procedure and the significance level for variables for removal from the model was set at 0.4. The Hosmer and Lemeshow test was used to assess the goodness-of-fit of the final models.The following variables were included in all Models: gender of parents (male = 0; female = 1), age of parent (continous, in years), gender of children (male = 0; female = 1), age of children (continous, in years), educational level of at least one parent (others = 0; college degree or higher = 1), number of cohabiting (continous), at least one parent who is a health care professional (no = 0; yes = 1), socio-economic status (three categories: low = 1; intermediate = 2; high = 3), days lost from school due to illness (no = 0; yes = 1), chronic conditions of children (0 = none; 1 = ≥1), health problem in the last year (no = 0; yes = 1), parent’s perception of health problem of child (others = 0; serious = 1), at least one hospital admission in the last year (no = 0; yes = 1). The variable at least one specilist visit in the last year (no = 0; yes = 1) was included in Model 1 and in Model 4. The variable emergency department visits in the last year (no = 0; yes = 1) was included in Model 1, in Model 2 and in Model 3. The variable at least one preventive care visit in the last year (no = 0; yes = 1) was included in Model 1 and Model 4. Finally,the variable utilization of the GP or FP in the last year (no = 0; yes = 1) was included in Model 2, in Model 3 and in Model 4 (see Additional file 2).

The results of the multivariate regression models have been expressed as odds ratios (ORs) and 95% confidence intervals (CIs) with a statistically significant level of *p*-value ≤0.05. Statistical analyses were performed using Stata version 10.1 software [27].

## Results

### Participants’ characteristics

In total, 1198 questionnaires were distributed and 891 parents agreed to participate in the survey with a response rate of 74.4%. 231 participants were parents of children selected from the three primary schools and 660 were parents of the students attending the nine secondary schools. The main characteristics of the children and parents are described in Table 1. More than two-thirds of parents were female (77.8%) and married (87.8%), the average age was 43.4 years. More than half of the sample had at least one child (61.2%) and approximately half of the sample had completed the high school education (49.3%). Regarding the children’s characteristics, only 9.6% had at least one parent who was a health care professional. The majority were female (59.7%), the average age was 12.6 years, approximately one in five had at least one chronic condition and 43.7% had had days absent from school due to health problems in the last year. Moreover, almost all parents (93.6%) considered the health status of their children to be at least good.

**Table 1:** Main characteristics of the children and parents

	Total	
	(*n* = 891)	
Parents	N	%
*Gender*		
Male	198	22.2
Female	693	77.8
*Age (years)*	43.4 ± 6.4(21–71)^a^	
*Marital status*		
Married	783	87.8
Other	108	12.2
*Educational level*		
Primary school or less	20	2.3
Middle school	166	18.6
High school	439	49.3
Baccalaureate/graduate degree	266	29.8
*At least one parent who is a health care professional*		
No	805	90.4
Yes	86	9.6
*Number of children*		
1	107	12
2	545	61.2
> 2	239	26.8
*Number of cohabiting*	3.3 ± 1.1(1–10)^a^	
Children		
*Gender*		
Male	359	40.3
Female	532	59.7
*Age (years)*	12.6 ± 3.5(5–18)^a^	
5–10	231	25.9
11–14	353	39.6
15–18	307	34.5
*Chronic conditions*		
None	744	83.5
At least one	147	16.5
*Health problems in the last year*		
No	602	67.6
Yes	289	32.4
*Parent’s perception of health problem*		
Very slight	301	33.8
Slight	198	22.2
Moderate	352	39.5
Serious	40	4.5
*Having day lost from school due to health problems*		
No	502	56.3
Yes	389	43.7

^a^Mean ± standard deviation (range)

Number for each item may not add up to total number of study population due to missing value

### Utilization of health care services in the previous 12 months

The proportion of utilization of at least one health care service (FP, GP, specialist, ED, hospital admission) by children in the previous 12 months was 91.6% and is detailed in Table 2.

**Table 2:** Utilization of health care services of overall sample according to the different age groups

	Total		5–10 years		11–14 years		15–18 years	
	(*n* = 891)		(*n* = 231)		(*n* = 353)		(*n* = 307)	
	N	%	N	%	N	%	N	%
*Utilization of the general practitioner/family pediatrician*	608	68.2	186	80.5	235	66.6	187	60.9
*Medical specialist’s visits in the last year*	590	66.2	137	59.3	247	69.9	206	67.1
Ophthalmology^a^	405	68.6	90	65.7	176	71.3	139	67.5
Dermatology^a^	179	30.3	36	26.3	70	28.4	73	35.4
Ortopedic^a^	158	26.8	35	25.5	71	28.7	52	25.2
Otolaryngology^a^	122	20.6	48	35.1	46	18.6	32	15.7
*Preventive care visits in the last year*	488	54.8	133	57.6	211	59.8	144	46.9
Ophthalmology^b^	337	69.1	76	57.1	151	71.6	110	76.4
Ortopedic^b^	74	15.2	15	11.3	38	18.1	17	11.8
Otolaryngology^b^	64	13.1	25	18.8	28	13.3	11	7.6
Dermatology^b^	51	10.5	9	6.8	22	10.4	20	13.9
*Dental visits in the last year*	540	60.6	132	57.1	226	64.1	182	59.3
*Preventive care dental visits in the last year*	72	8.1	8	3.5	21	5.9	43	14.1
*Emergency department visits in the last year*	114	12.8	32	13.9	38	10.8	44	14.3
*Hospitalizations in the last year*	42	4.7	10	4.3	15	4.2	17	5.5

^a^Only for children who have at last one specialist visit in the last year (*n* = 590)

^b^Only for children who have at last one preventive care visit in the last year (*n* = 488)

32.4% of respondents reported that their child had had a health problem in the last year and 68.2% reported at least one visit to the GP or FP in the previous 12 months. Among these parents, 54.7% were going to their GP because of confidence in their professional abilities and 32.2% for the ease utilization. Among the parents who had not gone preliminarily to their GP or FP, the main reasons were the long waiting time for the utilization (42.2%) and the greater chance of having a more appropriate diagnosis from a specialized ambulatory center (32.7%). The results of the multiple logistic regression analysis revealed that the utilization of the GP or FP in the last year was significantly higher amongst children who had experienced a health problem in the last year (OR = 1.7; CI 95% = 1.17–2.48), amongst those whose parents perceived their health problem as serious (OR = 4.39; CI 95% = 1.29–14.92), among children who had been absent from school due to health problem (OR = 1.67; CI 95% = 1.19–2.33) and amongst those who had had at least one specialist (OR = 1.47; CI 95% = 1.01–2.14) and preventive care visit in the last year (OR = 2.35; CI 95% = 1.64–3.38). However, the utilization of the GP or FP was significantly lower in younger children (OR = 0.91; CI 95% = 0.87–0.0.96) and amongst those who had at least one parent who was a health care professional (OR = 0.35; CI 95% = 0.21–0.6) (Table 3).

**Table 3:** Results of multivariate regression analysis to explore the characteristics associated with the use of the health care services

Variable	OR	SE	95% CI	*p* value
Model 1. General practitioners or family pediatricians utilization in the last year				
Log likelihood = −477.2 χ^2^=145.66 (12df), *p* < 0.0001				
At least one parent who is a health care professional	0.35	0.09	0.21–0.6	<0.001
At least one preventive care visit in the last year	2.35	0.43	1.64–3.38	<0.001
Age of children	0.91	0.02	0.87–0.96	0.001
Days lost from school due to illness	1.67	0.28	1.19–2.33	0.003
Health problems in the last year	1.7	0.33	1.17–2.48	0.005
Perception of health problem by the parent	4.39	2.74	1.29–14.92	0.018
At least one medical specialist’s visits in the last year	1.47	0.28	1.01–2.14	0.041
Educational level	1.37	0.25	0.95–1.97	0.09
At least one hospital admission in the last year	2.35	1.24	0.83–6.64	0.106
Gender of children	0.98	0.01	0.95–1.01	0.187
Chronic conditions	1.39	0.32	0.88–2.19	0.158
Emergency department visits in the last year	1.31	0.35	0.77–2.21	0.312
Model 2. Specialist utilization in the last year				
Log likelihood = −528.5 χ^2^=68 (10df), *p* < 0.0001				
At least one visit to general practitioners or family pediatricians in the last year	2.36	0.38	1.72–3.24	<0.001
Gender of children	1.86	0.28	1.38–2.51	<0.001
Emergency department visits in the last year	2.06	0.53	1.24–3.43	0.005
Age of children	1.05	0.02	0.99–1.1	0.054
Age of parent	1.02	0.01	0.99–1.05	0.134
Health problems in the last year	1.28	0.21	0.93–1.78	0.138
Number of children				
1	1^a^			
> 2	0.84	0.14	0.61–1.17	0.301
At least one hospital admission in the last year	0.69	0.26	0.33–1.46	0.343
Educational level	1.16	0.19	0.84–1.61	0.356
Gender of parent	1.18	0.22	0.82–1.69	0.382
Model 3. Preventive care visits in the last year				
Log likelihood = −564.68 χ^2^=85.75 (11df), *p* < 0.0001				
At least one visit to general practitioners or family pediatricians in the last year	2.85	0.45	2.09–3.88	<0.001
Gender of children	1.72	0.25	1.29–2.31	<0.001
Educational level	1.5	0.27	1.06–2.13	0.023
Age of children	0.95	0.02	0.91–0.99	0.029
Age of parent	1.03	0.01	1.01–1.06	0.033
Number of cohabiting	1.13	0.08	0.98–1.3	0.086
Chronic conditions	1.3	0.26	0.88–1.92	0.181
Socio-economic status				
low	1^a^			
medium	1.2	0.2	0.87–1.66	0.260
Gender of parent	1.22	0.22	0.86–1.74	0.266
Number of children				
1	1^a^			
2	1.15	0.17	0.85–1.55	0.354
Health problems in the last year	0.87	0.14	0.64–1.19	0.394
Model 4. Emergency department visits in the last year				
Log likelihood = −564.68 χ^2^=85.75 (11df), *p* < 0.0001				
At least one hospital admission in the last year	8.79	3.27	4.24–18.22	<0.001
Educational level	0.4	0.11	0.23–0.69	0.001
At least one medical specialist’s visits in the last year	2.45	0.69	1.41–2.26	0.001
Chronic conditions	2.09	0.53	1.27–3.46	0.004
Health problems in the last year	1.71	0.39	1.09–2.66	0.018
Age of parent	0.97	0.01	0.94–1.01	0.096
Socio-economic status				
low	1^a^			
medium	0.71	0.17	0.45–1.13	0.151
Gender of children	0.73	0.16	0.47–1.12	0.156
Perception of health problem by the parent	0.47	0.26	0.16–1.39	0.175
Number of children				
1	1^a^			
2	0.77	0.18	0.49–1.21	0.258
Number of cohabiting	1.11	0.1	0.91–1.35	0.288
At least one preventive care visit in the last year	0.78	0.19	0.49–1.25	0.31

^a^Reference category

Regarding the utilization of a specialist, 66.2% of children had had at least one visit to a specialist in the last year with a mean of 2.7 and more than half (60.6%) had had at least one dental visit with a mean of 4.3. The medical specialist visits more frequently reported (excluding dental visits) were visits to ophthalmologist (68.6%), dermatology (30.4%), orthopedic (26.8%) and otolaryngology (21.4%) specialists. The utilization of a specialist (excluding dental visits) was more likely among female children (OR = 1.86; CI 95% = 1.38–2.51), and amongst those who had had at least one visit to the GP or FP (OR = 2.36; CI 95% = 1.72–3.24) and emergency department in the last year (OR = 2.06; CI 95% = 1.24–3.43) (Table 3).

More than half of the children (54.8%) had had preventive care visits in the last year. Whereas only 8.1% of children had had preventive care dental visits. The more frequent preventive care visits for children reported by parents (excluding dental visits) were visits to ophthalmologist (69.1%), orthopedic (15.2%), otolaryngology (13.1%) and dermatology (10.5%) specialists. The model built to evaluate the variables associated with the use of preventive health care services (excluding preventive dental visits) in the last year showed that this use was significantly higher amongst female children (OR = 1.72; CI 95% = 1.29–2.31), among those who had GP or FP utilization (OR = 2.85; CI 95% = 2.09–3.88), amongst younger children (OR = 0.95; CI 95% = 0.91–0.99), amongst children whose parent was older in age (OR = 1.03; CI 95% = 1.01–1.06) and amongst children whose parent had a college degree or higher (OR = 1.5; CI 95% = 1.06–2.13) (Table 3).

The proportion of emergency department visits and hospital admissions of children reported were 12.8% and 4.7% respectively. The utilization of the emergency department was significantly more likely amongst children who had experienced a health problem in the last year (OR = 1.71; CI 95% = 1.09–2.66), amongst those who had chronic conditions (OR = 2.09; CI 95% = 1.27–3.46), amongst those who had at least one specialist visit in the last year (OR = 2.45; CI 95% = 1.41–2.26) and amongst the children who had had hospital admissions in the last year (OR = 8.79; CI 95% = 4.24–18.22). Moreover, the utilization of the emergency department was lower amongst children whose parent had a college degree or higher (OR = 0.4; CI 95% = 0.23–0.69) (Table 3).

## Discussion

To our knowledge, this survey is the first study carried out in Italy that evaluated the use of health care services, and the relative predictors, by children and adolescents regarding both the treatment and the prevention of diseases. The findings of this survey are difficult to compare with those of other studies conducted worldwide due the different characteristics of the populations sampled, methodologies, study period and the differences of the health care services in these countries.

In this study almost all children (91.6%) had had at least one utilization of health care services in the previous year. This result is in accordance with a value (92.5%) founded in Spain [18] but is higher than that found in a cross-sectional survey conducted among a large sample of children and adolescents from 11 European countries (65.4%) [15] and in a study among children aged 0 to 17 years in the USA (72%) [19]. Of more interest is that only two-thirds of the sample had had at least one visit to their GP or FP in the last year. This value is very low considering that Italy is a country with a system of universal health coverage and the utilization of the FP or GP is provided free of charge to all members of public. Moreover, among the parents who had not gone preliminarily to their GP or FP, the main reasons were the long waiting time for the utilization and the greater chance of having a more appropriate diagnosis from a specialist. These results point to the need for other sudies to investigate more thoroughly the non-medical mechanism of the health services use in order to provide important information to policy makers. However, the value of the utilization of the GP or FP in our survey is in accordance with the values found in a study conducted in Germany (68.3%) among younger adolescents [28] and in a survey conducted in Canada among adolescent and young adults. The latter found 67.1% of adolescents between 12 to 14 year olds and 69.8% between 15 to 19 year olds had used their family pediatrician in the last 12 months [29].

A key objective of this study was to investigate the frequency of preventive care visits among children and adolescents. In our sample, 54.2% of children had had at least one preventive care visit in the last year. These values are low and alarming since childhood and adolescence are key periods for the prevention and early treatment of health issues. Also, several institutions have developed guidelines and recommendations for the prevention of diseases and the protection of the health of infants, children and adolescents through the improvement of the quality of health care services, the continuity of care and access to regular preventive visits [30–32]. In other studies the proportions of preventive care visits for children and adolescents were higher than those observed in our study. In particular, the rates of preventive medical care visits were 88.2%, 68.9% and 69% respectively in early childhood, middle childhood and adolescence [23], 88.3% among children aged 1 to 17 years [17], 72% among children of married parents [14] and between 43 and 81.2% among a sample of adolescents in a study which compared three surveys [26]. Conversely, the proportion of children who had had at least one preventive care visit was lower in previous studies conducted in the USA [19, 20, 24].

Very low in this survey was the proportion of children who had had preventive care dental visits in the previous 12 months. Indeed, only 8.1% of children had at least one preventive dental visit. This worrying result can be explained by the fact that in Italy free public utilization of dental care is very difficult and that there are few public health facilities that provide dental services. Indeed, the value that has been observed is much lower than those reported in other studies, since a range from 38.8% to 82.6% of children had preventive dental care visit in the US [14, 17, 33, 34], 35.4% in Jordan [35], 35.3% of 12 year-olds and 20.2% of 18 year-olds children in China [36] and 12.5% in India [37].

In our study only 12.8% of the children had had emergency department visits in the last year. This observed value is lower than those reported in other studies, such as in two studies conducted in the US where the frequency of reported ED visits was respectively 29% and 19% [20, 21]. In a study conducted in the UK, one in three children had at least one utilization of the ED [22] and our observed value is identical to the levels of 12.8% of ED use among children reported in the USA [13] and to 12% reported in an already cited study [19].

Four multivariate regression models have been developed to evaluate the association of the characteristics of parents and children with the use of different health care services. Several characteristics including age and sex of children, educational level of parents, health status of children and the utilization of health care services were significantly associated with the different outcomes of interest. In particular, the preventive care visit was significantly more likely among children who had parents with a higher educational level. This finding has been found in previous studies [14]. Instead, in this survey a higher parental education level was inversely associated with the use of the emergency department. More interestingly in this study is that the use of the ED was strongly associated to chronic conditions and to the use of the other health care facilities such as specialist visits and hospital admission in the last year. These results are probably due to the fact that the children with chronic disorders are more in need of the utilization of the health care services.

This study has some limitations that should be considered in evaluating the results. Firstly, this study is a cross-sectional survey where the temporal direction of the association between the outcomes and the independent variables cannot be determined. Secondly, since we had asked parents to indicate the use of the health services for their children in the last 12 months, a recall bias may have occurred that could lead to underestimate or overestimate the use of health care services. The recall bias may affect the reliability of the data in particular for the responses regarding the reasons of consultation and the numbers of previous visits by GP or FP and the utilization of health care services for children and adolescents with chronic conditions. Finally, we have considered the use of health services in an area of the Campania Region which may be different from that of analogous samples in other Italian regions, in particular regarding the utilization of the preventive services. Therefore, although we cannot exclude that our results pertain only to our area, it is reasonable to assume that our sample may be analogous to that of other regions of southern Italy. To get a clearer framework regarding the use of health services by children and adolescents in Italy, we strongly recommend the replication of the study in the regions of the north of the country.

## Conclusion

In conclusion, this study provides relevant information on the use of health services by children and adolescents in Southern Italy. Relevants findings were the low value of the preventive visits rates and the higher educational level of parents as one of main determinants of using the preventive health services while no association was observed between the parent’s socio-economic status and preventive care utilization. These results highlight the need of educational interventions for parents and adolescents in order to increase the utilization of preventive health services. Moreover, parents’ responses have also revealed some non-medical reasons for which they chose to use the services and, since this study did not evaluate the access to services, future studies should addressed this topic to provide to policy makers and physicians information to make health care services more accessible, usable and efficient.

## Additional files

Additional file 1: Questionnaire: Self-administered questionnaire used for the survey. (DOCX 19 kb)

Additional file 2: Regression Modeling Strategy: Modeling Strategy used to build the multivariate regression final models. (DOCX 36 kb)

## Abbreviations

CI: Confidence interval
ED: Emergency department
FP: Family pediatrician
GP: General practitioner
NCDs: Noncommunicable diseases
OR: Odds ratios.
