# Supplementary material for: Evaluation of the use of health care services for non-communicable disease and prevention by children and adolescents in south Italy
Source: BMC Health Serv Res. 2017 Aug 4;17:532. doi: 10.1186/s12913-017-2489-4 (PMC5545043; doi:10.1186/s12913-017-2489-4)
Supplement: Supplementary file 1 — Questionnaire: Self-administered questionnaire used for the survey. (DOCX 19 kb) [file 12913_2017_2489_MOESM1_ESM.docx]

**QUESTIONNAIRE**

**Section A.**

**This section is designed to gather information about your socio-demographic characteristics.**

**A1.** Gender  Male  Female **A2.** How old were you on your last birthday? ____________

**A3.** How old were your partner on her last birthday? ______________

**A4.** What is your marital status?  Married  Single (never married)  Other _______________

**A5.** What is your occupation?________________

**A6.** What is your highest education level? _____________________

**A7.** What is your partner occupation? _____________________

**A8.** What is the highest education level of your partner? _____________________

**A9.** How many persons are there in your household? (not counting you) _____________

**A10.** How many children do you have? ______

**Section B.**

**This section is designed to gather information about socio-demographical and clinical characteristics of the child who brought you the questionnaire.**

**B1.** Gender  Male  Female **B2.** How old was your child on her last birthday? _________

**B3.** Has your child been absent from school for a health problem in the last year? □ no □ yes, how many times? _______________

Specify the reason for each time: 1______________________2 ____________________________

3 ________________________ 4 ______________________5 ____________________________

**B4.** Does your child suffer from any chronic diseases (eg. diabetes, asthma, allergies, hereditary disease, etc.)? □ no □ yes, which ones?______________________________________

**B5.** How would you classify your child current health status?

□ bad □ fair □ good □ very good □ excellent

**Section C.**

**This section is designed to gather information about the use of healthcare services by children and adolescents.**

**C1.** In the last 12 months your child has had a health problem? □ no □ yes

**C2.** How did you perceive your child health problem?

□ very mild □ mild □ moderate □ severe □ serious

**C3.** What did you do when your child has had a health problem in the last 12 months?

(even more than one answer) □ I went to the General Practitioner □ I asked for a phone consultation to the General Practitioner □ I went to the Family Pediatrician □ I asked for a phone consultation to the Family Pediatrician □ I've tried to cure it with drugs that I had at home□ I went to family/friends □ I went to the emergency department □ I went to a specialist □ Other (please specify) _______________________________________________________________________________

**C4.** Why did you go to the General Practitioner/Family Pediatrician (only if you went to the General Practitioner/Family Pediatrician) ?_____________________________________________

**C5.** Why did not you go to the General Practitioner/Family Pediatrician for the health problem of your child (only if you did not go preliminary to the General Practitioner/Family Pediatrician)?

________________________________________________________________________________

**C6.** Has your child been visited by the General Practitioner/Family Pediatrician in the last year?

 no  yes, how many times? _________

Specify the reason for each time: 1______________________2 ____________________________

3 ________________________4 ______________________5 _____________________________

**C7.** Has your child been visited by the specialist in the last year?  no  yes, which specialist? (please specify)

| **Specialist** | **How many times?** | **For which reason?** |
| --- | --- | --- |
|  |  |  |
|  |  |  |
|  |  |  |
|  |  |  |
|  |  |  |

**C8.** Has your child been visited by physicians for preventive visits in the last year?  no  yes, which specialist? (please specify)

| **Physicians** | **How many times?** | **For which reason?** |
| --- | --- | --- |
|  |  |  |
|  |  |  |
|  |  |  |
|  |  |  |
|  |  |  |

**C9.** Does your child have had dental visits in the last year?

 no  yes, how many times? _________

Specify the reason for each time: 1______________________2 ____________________________

3 ________________________ 4 ______________________5 ____________________________

**C10.** Does your child have had visits to the emergency department in the last years?

 no  yes, how many times? _________

Specify the reason for each time: 1______________________2 ____________________________

3 ________________________ 4 ______________________5 ____________________________

**C11.** Does your child have had hospitalizations in the last years?  no  yes, how many times? __

Specify the reason for each time: 1______________________2 ____________________________

3 ________________________ 4 ______________________5 ____________________________
