# Supplementary material for: Evaluation of the use of health care services for non-communicable disease and prevention by children and adolescents in south Italy
Source: BMC Health Serv Res. 2017 Aug 4;17:532. doi: 10.1186/s12913-017-2489-4 (PMC5545043; doi:10.1186/s12913-017-2489-4)
Supplement: Supplementary file 2 — Regression Modeling Strategy: Modeling Strategy used to build the multivariate regression final models. (DOCX 36 kb) [file 12913_2017_2489_MOESM2_ESM.docx]

**REGRESSION MODELING STRATEGY**

**FULL MODEL 1**

| **Variable** | **OR** | **SE** | **95% CI** | ***p* value** | | |  |
| --- | --- | --- | --- | --- | --- | --- | --- |
| **Model 1.** General practitioners or family pediatricians utilization in the last year | | | | | | |  |
| Log likelihood=-476.57, χ^2^=146.92 (19df), p<0.0001 | | | | | | |  |
| Gender of parent | 1.09 | 0.21 | 0.74-1.61 | | 0.661 | | |
| Age of parent | 0.98 | 0.01 | 0.95-1.01 | | 0.233 | | |
| Gender of children | 0.87 | 0.14 | 0.63-1.21 | | 0.411 | | |
| Age of children | 0.91 | 0.02 | 0.87-0.96 | | 0.001 | | |
| Socio-economic status |  |  |  | |  | | |
| low | 1* |  |  | |  | | |
| medium | 0.98 | 0.21 | 0.65-1.48 | | 0.926 | | |
| high | 0.78 | 0.32 | 0.35-1.73 | | 0.546 | | |
| Educational level | 1.67 | 0.58 | 0.84-3.32 | | | 0.142 | |
| At least one parent who is a health care professional | 0.36 | 0.09 | 0.21-0.61 | | | <0.001 | |
| Number of cohabiting | 0.99 | 0.27 | 0.81-1.21 | | | 0.954 | |
| Number of children |  |  |  | | |  | |
| 1 | 1* |  |  | | |  | |
| 2 | 0.99 | 0.11 | 0.58-1.7 | | | 0.976 | |
| >2 | 1.07 | 0.39 | 0.52-2.18 | | | 0.851 | |
| Chronic conditions | 1.34 | 0.32 | 0.85-2.13 | | | 0.209 | |
| Health problems in the last year | 1.7 | 0.33 | 1.17-2.48 | | | 0.006 |  |
| Perception of health problem by the parent | 4.47 | 2.8 | 1.31-15.29 | | | 0.017 |  |
| Emergency department visits in the last year | 1.29 | 0.34 | 0.76-2.18 | | | 0.345 |  |
| At least one hospital admission in the last year | 2.37 | 1.25 | 0.84-6.68 | | | 0.103 |  |
| At least one preventive care visit in the last year | 2.37 | 0.44 | 1.64-3.41 | | | <0.001 |  |
| At least one medical specialist’s visits in the last year | 1.49 | 0.29 | 1.02-2.17 | | | 0.037 |  |
| Days lost from school due to illness | 1.66 | 0.28 | 1.19-2.33 | | | 0.003 |  |

*Reference category

**BACKWARD ELIMINATION** (The significance level for variables for removal from the model was set at 0.4)

| **Variable** | ***p* value** |
| --- | --- |
| Gender of parent | 0.670 |
| Gender of children | 0.479 |
| Socio-economic status |  |
| medium | 0.923 |
| high | 0.532 |
| Number of cohabiting | 0.939 |
| Number of children |  |
| 2 | 0.975 |
| >2 | 0.71 |

**FINAL MODEL 1**

| **Variable** | **OR** | **SE** | **95% CI** | ***p* value** | | |  |
| --- | --- | --- | --- | --- | --- | --- | --- |
| **Model 1.** General practitioners or family pediatricians utilization in the last year | | | | | | |  |
| Log likelihood=-477.2 χ^2^=145.66 (12df), p<0.0001 | | | | | | |  |
| At least one parent who is a health care professional | 0.35 | 0.09 | 0.21-0.6 | | <0.001 | | |
| At least one preventive care visit in the last year | 2.35 | 0.43 | 1.64-3.38 | | <0.001 | | |
| Age of children | 0.91 | 0.02 | 0.87-0.96 | | 0.001 | | |
| Days lost from school due to illness | 1.67 | 0.28 | 1.19-2.33 | | 0.003 | | |
| Health problems in the last year | 1.7 | 0.33 | 1.17-2.48 | | | 0.005 | |
| Perception of health problem by the parent | 4.39 | 2.74 | 1.29-14.92 | | | 0.018 | |
| At least one medical specialist’s visits in the last year | 1.47 | 0.28 | 1.01-2.14 | | | 0.041 | |
| Educational level | 1.37 | 0.25 | 0.95-1.97 | | | 0.09 | |
| At least one hospital admission in the last year | 2.35 | 1.24 | 0.83-6.64 | | | 0.106 |  |
| Gender of children | 0.98 | 0.01 | 0.95-1.01 | | | 0.187 |  |
| Chronic conditions | 1.39 | 0.32 | 0.88-2.19 | | | 0.158 |  |
| Emergency department visits in the last year | 1.31 | 0.35 | 0.77-2.21 | | | 0.312 |  |

**Hosmer and Lemeshow goodness-of-fit test** χ^2^=8.88 (*p*=0.352)

**FULL MODEL 2**

| **Variable** | **OR** | **SE** | **95% CI** | ***p* value** |  |
| --- | --- | --- | --- | --- | --- |
| **Model 2.** Specialist utilization in the last year | | | | |  |
| Log likelihood=-526.5, χ^2^=72.2 (18df), p<0.0001 | | | | |  |
| Gender of parent | 1.19 | 0.22 | 0.82-1.72 | 0.349 | |
| Age of parent | 1.02 | 0.01 | 0.99-1.05 | 0.138 | |
| Gender of children | 1.84 | 0.28 | 1.36-2.49 | <0.001 | |
| Age of children | 1.06 | 0.03 | 1.01-1.11 | 0.029 | |
| Socio-economic status |  |  |  |  | |
| low | 1* |  |  |  | |
| medium | 1.38 | 0.27 | 0.93-2.04 | 0.107 | |
| high | 1.14 | 0.42 | 0.55-2.33 | 0.725 | |
| Educational level | 1.21 | 0.37 | 0.65-2.21 | 0.555 | |
| At least one parent who is a health care professional | 1.12 | 0.31 | 0.65-1.91 | 0.680 | |
| Number of cohabiting | 1.06 | 0.11 | 0.88-1.28 | 0.55 | |
| Number of children |  |  |  |  | |
| 1 | 1* |  |  |  | |
| 2 | 1.01 | 0.25 | 0.61-1.65 | 0.999 | |
| >2 | 0.79 | 0.27 | 0.41-1.53 | 0.490 | |
| Chronic conditions | 0.96 | 0.2 | 0.63-1.45 | 0.842 | |
| Health problems in the last year | 1.26 | 0.22 | 0.89-1.79 | 0.193 |  |
| Perception of health problem by the parent | 1.07 | 0.43 | 0.49-2.35 | 0.856 |  |
| Emergency department visits in the last year | 2.11 | 0.55 | 1.26-3.52 | 0.004 |  |
| At least one hospital admission in the last year | 0.68 | 0.26 | 0.32-1.44 | 0.313 |  |
| At least one visit to general practitioners or family pediatricians in the last year | 2.35 | 0.39 | 1.71-3.24 | <0.001 |  |
| Days lost from school due to illness | 1.12 | 0.18 | 0.81-1.54 | 0.483 |  |

*Reference category

**BACKWARD ELIMINATION** (The significance level for variables for removal from the model was set at 0.4)

| **Variable** | ***p* value** |
| --- | --- |
| Perception of health problem by the parent | 0.856 |
| At least one parent who is a health care professional | 0.671 |
| Chronic conditions | 0.844 |
| Socio-economic status |  |
| medium | 0.856 |
| high | 0.719 |
| Number of cohabiting | 0.526 |
| Number of children |  |
| 2 | 0.999 |
| Days lost from school due to illness | 0.457 |

**FINAL MODEL 2**

| **Variable** | **OR** | **SE** | **95% CI** | ***p* value** |  |
| --- | --- | --- | --- | --- | --- |
| **Model 2.** Specialist utilization in the last year | | | | |  |
| Log likelihood=-528.5 χ^2^=68 (10df), p<0.0001 | | | | |  |
| At least one visit to general practitioners or family pediatricians in the last year | 2.36 | 0.38 | 1.72-3.24 | <0.001 | |
| Gender of children | 1.86 | 0.28 | 1.38-2.51 | <0.001 | |
| Emergency department visits in the last year | 2.06 | 0.53 | 1.24-3.43 | 0.005 | |
| Age of children | 1.05 | 0.02 | 0.99-1.1 | 0.054 | |
| Age of parent | 1.02 | 0.01 | 0.99-1.05 | 0.134 | |
| Health problems in the last year | 1.28 | 0.21 | 0.93-1.78 | 0.138 | |
| Number of children |  |  |  |  | |
| 1 | 1* |  |  |  | |
| >2 | 0.84 | 0.14 | 0.61-1.17 | 0.301 | |
| At least one hospital admission in the last year | 0.69 | 0.26 | 0.33-1.46 | 0.343 | |
| Educational level | 1.16 | 0.19 | 0.84-1.61 | 0.356 |  |
| Gender of parent | 1.18 | 0.22 | 0.82-1.69 | 0.382 |  |

**Hosmer and Lemeshow goodness-of-fit test** χ^2^=8.12 (*p*=0.422)

**FULL MODEL 3**

| **Variable** | **OR** | **SE** | **95% CI** | ***p* value** |  |
| --- | --- | --- | --- | --- | --- |
| **Model 3.** Preventive care visits in the last year | | | | |  |
| Log likelihood=-564.11, χ^2^=86.9 (18df), p<0.0001 | | | | |  |
| Gender of parent | 1.23 | 0.22 | 0.87-1.76 | 0.243 | |
| Age of parent | 1.03 | 0.01 | 1.01-1.05 | 0.031 | |
| Gender of children | 1.72 | 0.25 | 1.29-2.31 | <0.001 | |
| Age of children | 0.95 | 0.02 | 0.91-0.99 | 0.028 | |
| Socio-economic status |  |  |  |  | |
| low | 1* |  |  |  | |
| medium | 1.38 | 0.22 | 0.78-1.65 | 0.502 | |
| high | 0.83 | 0.29 | 0.41-1.67 | 0.599 | |
| Educational level | 1.64 | 0.5 | 0.91-2.97 | 0.099 | |
| At least one parent who is a health care professional | 1.35 | 0.29 | 0.68-1.69 | 0.627 | |
| Number of cohabiting | 1.14 | 0.1 | 0.95-1.37 | 0.142 | |
| Number of children |  |  |  |  | |
| 1 | 1* |  |  |  | |
| 2 | 1.1 | 0.27 | 0.68-1.78 | 0.696 | |
| >2 | 0.91 | 0.29 | 0.48-1.72 | 0.776 | |
| Chronic conditions | 1.27 | 0.25 | 0.86-1.89 | 0.224 | |
| Health problems in the last year | 0.86 | 0.14 | 0.62-1.2 | 0.389 |  |
| Perception of health problem by the parent | 0.91 | 0.32 | 0.45-1.83 | 0.787 |  |
| Emergency department visits in the last year | 1.09 | 0.25 | 0.7-1.7 | 0.688 |  |
| At least one hospital admission in the last year | 0.89 | 0.32 | 0.44-1.79 | 0.751 |  |
| At least one visit to general practitioners or family pediatricians in the last year | 2.86 | 0.46 | 2.08-3.92 | <0.001 |  |
| Days lost from school due to illness | 1.08 | 0.17 | 0.79-1.46 | 0.614 |  |

*Reference category

**BACKWARD ELIMINATION** (The significance level for variables for removal from the model was set at 0.4)

| **Variable** | ***p* value** |
| --- | --- |
| Perception of health problem by the parent | 0.787 |
| At least one parent who is a health care professional | 0.613 |
| Emergency department visits in the last year | 0.733 |
| At least one hospital admission in the last year | 0.713 |
| Socio-economic status |  |
| high | 0.616 |
| Number of children |  |
| >2 | 0.779 |
| Days lost from school due to illness | 0.629 |

**FINAL MODEL 3**

| **Variable** | **OR** | **SE** | **95% CI** | ***p* value** |  |
| --- | --- | --- | --- | --- | --- |
| **Model 3 .** Preventive care visits in the last year | | | | |  |
| Log likelihood=-564.68 χ^2^=85.75 (11df), p<0.0001 | | | | |  |
| At least one visit to general practitioners or family pediatricians in the last year | 2.85 | 0.45 | 2.09-3.88 | <0.001 | |
| Gender of children | 1.72 | 0.25 | 1.29-2.31 | <0.001 | |
| Educational level | 1.5 | 0.27 | 1.06-2.13 | 0.023 | |
| Age of children | 0.95 | 0.02 | 0.91-0.99 | 0.029 | |
| Age of parent | 1.03 | 0.01 | 1.01-1.06 | 0.033 | |
| Number of cohabiting | 1.13 | 0.08 | 0.98-1.3 | 0.086 | |
| Chronic conditions | 1.3 | 0.26 | 0.88-1.92 | 0.181 | |
| Socio-economic status |  |  |  |  | |
| low | 1* |  |  |  | |
| medium | 1.2 | 0.2 | 0.87-1.66 | 0.260 | |
| Gender of parent | 1.22 | 0.22 | 0.86-1.74 | 0.266 | |
| Number of children |  |  |  |  | |
| 1 | 1* |  |  |  | |
| 2 | 1.15 | 0.17 | 0.85-1.55 | 0.354 | |
| Health problems in the last year | 0.87 | 0.14 | 0.64-1.19 | 0.394 |  |

*Reference category

**Hosmer and Lemeshow goodness-of-fit test** χ^2^=7.15 (*p*=0.520)

**FULL MODEL 4**

| **Variable** | **OR** | **SE** | **95% CI** | ***p* value** |  |
| --- | --- | --- | --- | --- | --- |
| **Model 4.** Emergency department visits in the last year | | | | |  |
| Log likelihood=-294.11, χ^2^=89.94 (19df), p<0.0001 | | | | |  |
| Gender of parent | 1.13 | 0.33 | 0.64-1.99 | 0.663 | |
| Age of parent | 0.97 | 0.02 | 0.93-1.01 | 0.145 | |
| Gender of children | 0.73 | 0.16 | 0.47-1.13 | 0.158 | |
| Age of children | 1.02 | 0.04 | 0.94-1.09 | 0.654 | |
| Socio-economic status |  |  |  |  | |
| low | 1* |  |  |  | |
| medium | 0.75 | 0.2 | 0.45-1.27 | 0.291 | |
| high | 1.42 | 0.92 | 0.39-5.07 | 0.599 | |
| Educational level | 0.3 | 0.17 | 0.09-0.94 | 0.039 | |
| At least one parent who is a health care professional | 1.23 | 0.55 | 0.52-2.94 | 0.634 | |
| Number of cohabiting | 1.14 | 0.13 | 0.9-1.45 | 0.26 | |
| Number of children |  |  |  |  | |
| 1 | 1* |  |  |  | |
| 2 | 0.7 | 0.25 | 0.35-1.41 | 0.325 | |
| >2 | 0.85 | 0.38 | 0.35-2.04 | 0.715 | |
| Chronic conditions | 2.07 | 0.53 | 1.25-3.42 | 0.005 | |
| Health problems in the last year | 1.67 | 0.39 | 1.05-2.66 | 0.031 |  |
| Perception of health problem by the parent | 0.46 | 0.25 | 0.15-1.35 | 0.158 |  |
| At least one hospital admission in the last year | 8.73 | 3.29 | 4.17-18.28 | <0.001 |  |
| At least one visit to general practitioners or family pediatricians in the last year | 1.26 | 0.33 | 0.75-2.11 | 0.379 |  |
| At least one preventive care visit in the last year | 0.78 | 0.19 | 0.48-1.25 | 0.303 |  |
| At least one medical specialist’s visits in the last year | 2.34 | 0.67 | 1.34-4.09 | 0.003 |  |
| Days lost from school due to illness | 0.98 | 0.23 | 0.62-1.56 | 0.949 |  |

*Reference category

**BACKWARD ELIMINATION** (The significance level for variables for removal from the model was set at 0.4)

| **Variable** | ***p* value** |
| --- | --- |
| Gender of parent | 0.664 |
| Age of children | 0.623 |
| At least one parent who is a health care professional | 0.641 |
| Socio-economic status |  |
| high | 0.628 |
| Number of children |  |
| >2 | 0.718 |
| Days lost from school due to illness | 0.452 |
| At least one visit to general practitioners or family pediatricians in the last year | 0.453 |

**FINAL MODEL 4**

| **Variable** | **OR** | **SE** | **95% CI** | ***p* value** | | |  |
| --- | --- | --- | --- | --- | --- | --- | --- |
| **Model 4.** Emergency department visits in the last year | | | | | | |  |
| Log likelihood=-564.68 χ^2^=85.75 (11df), p<0.0001 | | | | | | |  |
| At least one hospital admission in the last year | 8.79 | 3.27 | 4.24-18.22 | | <0.001 | | |
| Educational level | 0.4 | 0.11 | 0.23-0.69 | | 0.001 | | |
| At least one medical specialist’s visits in the last year | 2.45 | 0.69 | 1.41-2.26 | | 0.001 | | |
| Chronic conditions | 2.09 | 0.53 | 1.27-3.46 | | 0.004 | | |
| Health problems in the last year | 1.71 | 0.39 | 1.09-2.66 | | 0.018 | | |
| Age of parent | 0.97 | 0.01 | 0.94-1.01 | | 0.096 | | |
| Socio-economic status |  |  |  | |  | | |
| low | 1* |  |  | |  | | |
| medium | 0.71 | 0.17 | 0.45-1.13 | | 0.151 | | |
| Gender of children | 0.73 | 0.16 | 0.47-1.12 | | | 0.156 | |
| Perception of health problem by the parent | 0.47 | 0.26 | 0.16-1.39 | | | 0.175 | |
| Number of children |  |  |  | | |  | |
| 1 | 1* |  |  | | |  | |
| 2 | 0.77 | 0.18 | 0.49-1.21 | | | 0.258 | |
| Number of cohabiting | 1.11 | 0.1 | 0.91-1.35 | | | 0.288 |  |
| At least one preventive care visit in the last year | 0.78 | 0.19 | 0.49-1.25 | | | 0.31 |  |

*Reference category

**Hosmer and Lemeshow goodness-of-fit test** χ^2^=1.54 (*p*=0.992)
